# Development of training program for the Eye, Ear, Nose, and Throat emergency nurses in China based on core competency: a Delphi study

**DOI:** 10.1186/s12873-024-01032-8

**Published:** 2024-07-09

**Authors:** Yanqiu Hu, Fang Wang, Wenzhu Cao, Chunyi Gui

**Affiliations:** Department of Nursing, Eye & ENT Hospital, Fudan University, Shanghai, 200031 China

**Keywords:** EENT, Emergency, Training program, Delphi method

## Abstract

**Background:**

Nursing work in the Eye, Ear, Nose, and Throat (EENT) emergency department is highly specialised and faces significant challenges. Therefore, a high level of nursing competence is necessary for nurses. To develop core competencies, a systematic and standardised training program is required. This study aims to construct a standardised, systematic, and professional training program for nurses working in the EENT emergency department in China.

**Methods:**

Based on a literature review and semi-structured interviews, the training scheme draft was developed according to the theoretical framework of core competency for emergency nurses. From July 2023 to October 2023, a total of 21 experts including clinical experts, and nursing experts were selected to conduct 2 rounds of Delphi consultation to construct the training program for EENT emergency nurses.

**Results:**

The effective response rate for 2 rounds of expert consultation was 100%. The expert authority coefficient was 0.905, and Kendall’s W coefficients were found to be 0.359 and 0.340, respectively. The coefficients of variation for each item of the second round of expert consultation ranged from 0 to 0.19. The finalised training program for EENT emergency nurses consisted of 4 first-level indexes (training objectives, training management, training contents, and training assessment). The training objectives included 3 secondary indicators and 16 tertiary indicators. Training management included 5 secondary indicators and 8 tertiary indicators. Training contents included 4 secondary indicators and 16 tertiary indicators. Training assessment included 3 secondary indicators and 6 tertiary indicators.

**Conclusion:**

This study systematically and comprehensively explores the cultivation of nurses working in the EENT emergency department from the aspects of training objectives, training management, training contents, and training assessment. This training program is based on the theoretical framework of core competency standards for emergency nurses. It is in line with the actual needs of the clinic, and the training program is scientific and reliable, which can be promoted nationwide to provide a reference basis for the improvement of the training of emergency specialist nurses.

**Trial registration:**

Not applicable.

## Background

Emergency nurses as the primary providers of first aid play a pivotal role in EENT emergency care. Their involvement concerns clinical outcomes and the quality of emergency nursing care [1, 2]. In 2022, the National Health Commission of China issued the “National Nursing Career Development Plan (2021–2025)” [3], which emphasises the need to strengthen specialised training of emergency nurses and improve their professional competence. It was reported that most emergency nurses lack specialised skills and cannot correctly identify basic first aid measures for acute ENT emergencies [4], and their core competence still requires further improvement [5].

The core competence, originally derived from business management, is rooted in the transmission of experiential norms and values, collective learning, and mutual communication and participation within organisations [6]. Nursing core competence refers to the organic integration of systematic professional knowledge, skills, attitudes, and personal attributes that nurses acquire in a given nursing environment [7]. In 2010, the Emergency Nurses Association established competency standards for emergency nurses, including three dimensions: education, practice, and research [8]. In China, scholars such as Luo Fan [7] have constructed core competencies for specialised emergency nurses including six major competency dimensions: professional attitude, professional practice, critical thinking, management abilities, interpersonal communication skills, and professional development abilities. These competencies are closely aligned with the current status of emergency nurse education in China and provide a theoretical basis for the training and competency assessment of the subspecialised emergency nurses.

However, current research on emergency nursing in both domestic and international settings has mainly focused on pre-screening and triage standards [9, 10], triage management models [11], surveys of core competencies [12], improvements in training models [13, 14], and the assessment of pre-screening and triage skills [15]. Some general hospitals have developed systematic training programs for emergency nurses [16]. Some Asian and European countries have conducted studies on the training of specialist nurses in the relevant areas, including ophthalmic nursing [17–19], otolaryngology nursing [20, 21], and head and neck cancer care [22, 23]. Some studies have reported the effectiveness of short emergency courses for ENT nurses [24]. However, there is still no systematic training program specifically designed for the EENT emergency nurses. Therefore, this study will be guided by the core competencies of specialised emergency nurses and integrate these competency requirements into the training for EENT emergency nurses. The Delphi expert consultation method is an intuitive prediction technique that involves anonymous rounds of consultations to solicit expert opinions [25]. It is an appropriate method for the evaluation of clinical issues that remain unresolved and difficult to quantify [26]. Therefore, this study used the Delphi method to solicit opinions from the members of the expert group and constructed a standardised, systematic, and professional training program to enhance the clinical practice abilities of nurses in the EENT emergency department. This program provides a reference basis for the training of EENT emergency nurses in tertiary hospitals in China.

## Methods

### Formation of research team

The research team for this project consists of 5 members, including 2 nursing managers with the associate-senior title specialising in EENT nursing and nursing education, 1 Chief Physician in otolaryngology with a PhD, and 2 nurses with master’s degrees specialising in EENT nursing. Each member of the research team has specific responsibilities, including literature retrieval, conducting semi-structured interviews, compiling expert consultation forms, selecting experts, and performing data statistical analysis.

### Literature review

A literature review was conducted using keywords in both Chinese and English, including “EENT nurse/Ophthalmic nurse/Otolaryngology nurse/Emergency nurse/Nurse working in the emergency room/Emergency nurse practitioner, training program/training content, competence/core competence”. A literature search was conducted in Chinese databases (China National Knowledge Infrastructure, Wanfang Data, CQVIP, etc.) and the English database PubMed for relevant articles between 1 January 2017 and 30 June 2022. Two researchers independently conducted the literature search and selection, followed by a cross-checking process. In the event of disagreement, a third party was invited to discuss and resolve the differences. Initially, 1,186 relevant articles were identified, consisting of 597 English articles and 589 Chinese articles. Duplicate articles (69) were removed using NoteExpress software. Following the review of titles and abstracts, 329 articles were excluded as they did not align with the research objectives or were of poor quality. A total of 237 articles (191 in Chinese and 46 in English) were further reviewed in full text. Content analysis was performed on these articles, leading to the development of a preliminary framework for the training program, including training objectives, management, contents, and assessment components.

### Semi-structured interviews

Purposive sampling was used to select participants for one-on-one semi-structured interviews conducted between August and December 2022. The interviews aimed to ascertain the specific training needs and opinions of EENT emergency nurses. The inclusion criteria were as follows: (1) EENT emergency nursing managers, EENT emergency nursing educators, EENT emergency nurses, EENT emergency physicians, etc.; (2) Those working in the EENT emergency department for at least 3 years; (3) Those holding an associate degree or above; (4) Those who volunteered to participate. A total of 12 participants were included in the study, consisting of 2 EENT emergency nursing managers, 2 EENT emergency nursing educators, 3 EENT emergency nurses, and 5 EENT emergency physicians. Their ages ranged from 31 to 55 years, with 2 holding associate-senior titles, 7 holding intermediate titles, and 3 holding junior titles. The participants’ work experience ranged from 3 to 35 years. Four of the EENT emergency nurses held city-level specialised nurse certificates. The demographic data of the interviewees is presented in Table 1.

**Table 1:** Demographic data of the interviewees (*n* = 12)

Item	Project	Frequency	Proportion(%)
Gender	Female	8	66.67
	Male	4	33.33
Age(years)	30–34	7	58.33
	35–39	3	25.00
	≥ 40	2	16.67
Working experience (years)	<10	6	50.00
	10–15	4	33.33
	>15	2	16.67
Education background	Bachelor	7	58.33
	Doctorate	5	41.67
Job title	Junior level	3	25.00
	Intermediate level	7	58.33
	Associate-senior level	2	16.67
Job position	EENT Nursing manager	2	16.67
	EENT emergency nursing educator	2	16.67
	EENT emergency nurses	3	25.00
	EENT emergency physician	5	41.67

The interview outline was set up according to the relevant literature [27] and included the following questions: (1) What abilities and qualities do you think EENT emergency nurses should have? What abilities are currently lacking? (2) What are the responsibilities of EENT emergency nurses and how do they differ from those of emergency nurses in general hospitals? (3) What aspects of training for EENT emergency nurses should be included, and should training contents from general hospitals be incorporated? (4) Do you have any suggestions for training methods and assessment methods? (5) Do you have any suggestions regarding the admission criteria, training duration, and training instructors for EENT emergency nurses? (6) Do you have any opinions or suggestions regarding the training of EENT emergency nurses? Each interview lasted between 30 and 60 min. Following each interview, the Colaizzi content analysis method [28] was applied to analyse the interview data as follows: (1) Transcription: Within 24 h after the interview, the researcher prepared field notes for the current interview and transcribed the recording within 72 h, using English letters to represent the interviewees in the order of the interviews. The researcher conducted repeated listening to the recording, reviewed the interview notes and transcribed text, and analysed and organised the interview content based on research questions, extracting and labeling statements with significant meanings; (2) Coding: Coding was applied to recurring viewpoints; (3) Categorisation Analysis: The coded viewpoints were categorised using a classification approach for content analysis; (4) Description: Detailed and comprehensive descriptions were drafted, themes were refined, and verification was sought from the respondents; (5) Organisation: The data was organised into meaningful interpretations of the phenomenon.

### Preparation of expert consultation questionnaire

Based on previous relevant literature reviews and semi-structured interviews, the research team drafted an initial expert consultation questionnaire based on the core competencies of EENT emergency nurses, using the core competency assessment criteria for emergency nurses [7] as the theoretical basis. The questionnaire includes introductory instructions, basic information about the experts, and the main consultation questionnaire: (1) The introductory instructions provide a brief introduction to the background, objectives, and content of this study. It also clarifies the purpose of expert consultation, the tasks involved, and the method of completing the questionnaire, and emphasises the importance and significance of experts’ opinions for this research. (2) The experts’ basic information includes age, years of work experience, level of education, professional title, position, familiarity with the consultation questions, and a self-assessment of their judgment. (3) The main consultation questionnaire is an assessment form for the training program. It includes four primary indicators: training objectives, training management, training contents, and training assessment. Experts are asked to rate the importance of each indicator using a 5-point Likert scale. The score of “not important at all” was 1, “not very important” was 2, “generally important” was 3, “important” was 4, and “very important” was 5. Additionally, the table includes a section for expert comments which is designed to facilitate the experts in providing suggestions.

### Selection of experts

This research conducted two rounds of expert consultations from July 2023 to October 2023. The inclusion criteria for experts were as follows: (1) Expertise in emergency nursing or ophthalmology or otolaryngology; (2) Clinical teaching in emergency nursing or ophthalmology or otolaryngology or related fields; (3) Positions such as nursing managers, nursing educators, attending physicians; (4) At least a bachelor’s degree; (5) Intermediate professional title or higher; (6) at least of 5 years of work experience.

### Experts’ consultation

This study conducted two rounds of expert consultation. The consultation questionnaires were distributed via email from July 2023 to October 2023. Each round of expert consultation was limited to 2 weeks. After collecting the questionnaires of each round, the researcher organised the experts’ importance ratings and opinions for each item and calculated the mean importance rating and coefficient of variation. The research group discussed and screened all the indicators based and the expert consultation and recommendations, and attached the results to the next round of consultation. An importance rating ≥ 4.0 indicates a high level of importance, and a coefficient of variation < 0.2 indicates a high level of expert consensus. If either or both of these criteria were not met, the research team made decision through discussion to revise the indicators in the next round of consultation.

### Statistical methods

The data collection and statistical analysis were conducted using Excel 2016 and IBM SPSS Statistics 22.0 software. The authority and reliability of the results were determined by calculating the coefficient of expert enthusiasm, the degree of authority, the degree of coordination, and the degree of concentration. The coefficient of expert enthusiasm reflects the return rate of expert consultation letters, with a higher return rate indicating greater expert enthusiasm. The degree of expert authority is determined by the experts themselves and is represented by the coefficient of expert authority (Cr), which is calculated using two factors: the coefficient of judgment (Ca) and the familiarity of the experts with the content of the consultation (Cs), Cr = (Ca + Cs)/2. The degree of concentration of expert opinions is primarily influenced by the importance ratings of each item, the standard deviation, and the coefficient of variation. The coefficient of coordination of expert opinions is represented by Kendall’s W. A P value < 0.05 indicates a statistically significant difference.

## Results

### The results of the Delphi expert consultation

#### Basic expert information

The study included a total of 21 experts, consisting of 8 emergency nursing managers, 9 nursing educators, and 4 EENT medical specialists. Of the experts, 4 were male and 17 were female. The educational backgrounds of the participants varied, with 14 holding bachelor’s degrees, 3 holding master’s degrees, and 4 holding doctoral degrees. In terms of professional titles, 8 held senior titles, and 13 held intermediate titles. The experts’ ages ranged from 32 to 54 years, with an average work experience of (17.90 ± 8.79) years. The demographic data for the experts is shown in Table 2.

**Table 2:** Demographic data of the experts (*n*=21)

Item	Project	Frequency	Proportion(%)
Age(years)	30–34	3	14.29
	35–39	7	33.33
	40–44	5	23.81
	≥ 45	6	28.57
Working experience (years)	< 10	4	19.05
	10–15	4	19.05
	16–20	7	33.33
	> 20	6	28.57
Gender	Female	17	80.95
	Male	4	19.05
Education background	Bachelor	14	66.67
	Master	3	14.29
	Doctorate	4	19.05
Job title	Intermediate level	13	61.90
	Associate-senior level	7	33.33
	Senior level	1	4.76
Research field	EENT emergency nursing management and education	4	19.05
	Emergency nursing management and education	4	19.05
	EENT nursing and education	7	33.33
	Otorhinolaryngology nursing and education	2	9.52
	Ophthalmology	1	4.76
	Otorhinolaryngology	3	14.29

### Experts’ positive degree and Authority Coefficient

A total of 21 responses were received for both rounds of questionnaires, with a 100% response rate. In the first round, 10 experts provided modification suggestions, while in the second round, 2 experts provided further suggestions. The expert judgment coefficient (Ca) was 0.94, the expert familiarity coefficient (Cs) was 0.87, and the expert authority coefficient (Cr) was 0.905.

### The degree of Coordination and Concentration of Expert opinions

Following two rounds of expert consultation, the mean importance ratings for each item ranged from 4.0 to 5.0, and the coefficient of variation for each item ranged from 0 to 0.19. The coordination coefficient (Kendall’s W) for the first round of expert consultation was 0.359, and for the second round, Kendall’s W was 0.340, with a P value < 0.001. Further details can be found in Table 3.

**Table 3:** Results of the coordination degree of experts’ opinions

Round	Number of Indicators	Kendall’s W	${\varvec{x}}^{2}$	*P*
First	All Indicators (62)	0.359	87.667	0.014
	Primary Indicators (4)	0.094	5.933	0.115
	Secondary Indicators (15)	0.197	57.820	0.000
	Tertiary Indicators (43)	0.340	57.192	0.059
Second	All Indicators (65)	0.340	392.076	0.000
	Primary Indicators (4)	0.222	12.000	0.007
	Secondary Indicators (15)	0.287	72.208	0.000
	Tertiary Indicators (46)	0.350	283.302	0.000

### Summary of expert opinions

The first round of expert consultation included 4 primary indicators, 15 secondary indicators, and 43 tertiary indicators. Following this, 8 indicators were revised, and 5 new indicators were added based on the opinions and discussions held within the group, forming the second round of expert consultation questionnaire. The modifications made are outlined below:

(1) Training objectives: (1) Some experts considered that the concepts of “communication ability” and “teamwork abilities” were similar and could be merged into one indicator. After group discussion, the experts’ opinion was accepted, and the indicators were merged into “possessing communication, teamwork, and collaboration skills”. (2) Experts suggested that EENT emergency nurses should have a certain level of critical thinking skills to deal with urgent and complex clinical scenarios. After group discussion, the expert opinion was accepted, and “possess critical thinking skills” was added.

(2) Training management: (1) Some experts proposed that the work experience requirement for trainees should be extended and recommended changing it to “work experience ≥ 1 year”. This was accepted by the group. (2) Experts pointed out the need to include various forms of training and teaching methods, such as “workshops” or “simulation exercises”. After discussion, it was agreed that training formats are different from teaching methods. Consequently, expert opinions were partially accepted, and “teaching methods” were changed to “multimedia theoretical lectures, online video courses, special lectures, scenario simulations, one-on-one mentoring, live demonstrations, workshops, etc.”. (3) Some experts recommended extending the duration of the training, and after group discussion, it was accepted and modified to a training duration of 1–3 months. (4) Experts also suggested adding standards for trainers’ titles and educational backgrounds. After discussion, two indicators were added: “intermediate (attending physician, head nurse, etc.) or higher professional titles” and “engaging in departmental teaching work with teaching skills”.

(3) Training contents: (1) Some experts suggested that EENT emergency nurses should also be trained in the treatment and nursing care of some common medical and surgical emergencies, such as “gastrointestinal system: gastrointestinal bleeding, pancreatitis, neurological system: cerebral infarction, cerebral hemorrhage”. The group discussed that agreed that gastrointestinal disorders are extremely rare in EENT emergencies and that some neurological disorders may have similar symptoms as EENT diseases. Therefore, some expert opinions were partially adopted. (2) Some experts proposed the addition of “cricothyroid membrane puncture” to the list of specialised procedures and emergency nursing coordination projects for EENT. After discussion, the group concurred that cricothyroid membrane puncture is predominantly performed by physicians, instead of nurses. However, in certain instances of EENT emergencies, nurses may be required to assist physicians with tracheostomy procedures. Therefore, based on expert opinions, “assistance with percutaneous tracheostomy” was added. (3) Some experts suggested the inclusion of commonly used emergency equipment, such as “ventilators, electrical suction devices, infusion pumps, syringe pumps, oxygen cylinders, etc.” After group discussion, this expert opinion was incorporated. (4) Some experts recommended that the training contents should cover emergency nursing research to promote professional development. After discussion, the group adopted this suggestion and revised the indicator “Education in emergency nursing” to “Education and research in emergency nursing”. Additionally, the group added an indicator “Fundamentals of research in emergency nursing (including literature review, research design, and thesis writing methods, etc.)”.

(4) Assessment and evaluation: Some experts proposed that the assessment and evaluation of EENT emergency nurses should include not only theoretical and practical assessment but also “core competency assessment” in order to comprehensively evaluate the effectiveness of training. After group discussion, this expert opinion was accepted.

### Finalised training program for EENT Emergency nurses

Following two rounds of expert consultation, expert opinions converged, leading to the finalisation of the training program for EENT emergency nurses. The final draft included 4 primary indicators, 15 secondary indicators, and 46 tertiary indicators, as detailed in Table 4.

**Table 4:** EENT emergency nurse training program based on core competencies

Indicator Content	Importance Rating (Mean ± SD)	Coefficient of Variation
1. Training Objectives	5.00 ± 0.00	0.00
1.1 Knowledge Objectives	5.00 ± 0.00	0.00
1.1.1 Mastery of knowledge and nursing for common critical diseases in EENT and internal/external medicine	5.00 ± 0.00	0.00
1.1.2 Mastery of specialised emergency nursing techniques in EENT	5.00 ± 0.00	0.00
1.1.3 Mastery of pre-screening and triage methods in EENT emergencies	4.89 ± 0.32	0.07
1.1.4 Mastery of interpersonal communication methods in EENT emergencies	4.83 ± 0.38	0.08
1.1.5 Familiarity with relevant theoretical knowledge and teaching methods of clinical nursing education	4.33 ± 0.59	0.14
1.2 Ability Objectives	5.00 ± 0.00	0.00
1.2.1 Ability to provide emergency nursing care for critical patients with EENT conditions	5.00 ± 0.00	0.00
1.2.2 Ability to assess patient conditions accurately	5.00 ± 0.00	0.00
1.2.3 Ability to respond to unexpected emergencies	5.00 ± 0.00	0.00
1.2.4 Ability for communication, teamwork, and collaboration	4.94 ± 0.24	0.05
1.2.5 Ability to think critically	4.61 ± 0.50	0.11
1.2.6 Ability to provide health education to patients and their families	4.56 ± 0.51	0.11
1.2.7 Ability for clinical teaching and professional training	4.06 ± 0.73	0.18
1.3 Professionalism Objectives	4.89 ± 0.32	0.07
1.3.1 Responsibility, compassion, patience, and respect for patients and their families	4.89 ± 0.32	0.07
1.3.2 Compliance with laws, regulations, and related requirements with a spirit of prudence and diligence	5.00 ± 0.00	0.00
1.3.3 Good psychological qualities and self-regulation ability	4.56 ± 0.51	0.11
1.3.4 Strong sense of self-directed learning and continuous acquisition of new knowledge and skills in the field of emergency care	4.89 ± 0.32	0.07
2. Training Management	4.72 ± 0.46	0.10
2.1 Admission criteria for trainees	4.72 ± 0.46	0.10
2.1.1 Over a year of experience in the EENT department	4.44 ± 0.51	0.12
2.2 Trainer Qualifications	5.00 ± 0.00	0.00
2.2.1 Over 5 years of experience in emergency care/ophthalmology /otorhinolaryngology	4.94 ± 0.24	0.05
2.2.2 Hold a municipal-level specialist nurse certificate in emergency care	4.17 ± 0.79	0.19
2.2.3 Intermediate (attending physician, head nurse, etc.) or above	4.39 ± 0.78	0.18
2.2.4 Engaging in clinical teaching work with teaching abilities	4.72 ± 0.46	0.10
2.3 Training Methods	5.00 ± 0.00	0.00
2.3.1 Training methods: a combination of theoretical lectures and clinical practice, using a mix of online and offline formats	4.89 ± 0.32	0.07
2.4 Training Duration	4.50 ± 0.51	0.11
2.4.1 Training duration: 1–3 months	4.50 ± 0.51	0.11
2.5 Teaching Methods	4.94 ± 0.24	0.05
2.5.1 Teaching methods: multimedia theoretical lectures, online video courses, special lectures, scenario simulations, one-on-one mentoring, live demonstrations, workshops, etc.	4.83 ± 0.38	0.08
3. Training Contents	5.00 ± 0.00	0.00
3.1 Theoretical Knowledge of Emergency Medical Specialties	4.94 ± 0.24	0.05
3.1.1 Knowledge and nursing of common critical conditions in EENT: ophthalmology: ocular trauma (ocular chemical injury, electric ophthalmia, eye blast injury, etc.), retinal detachment, central retinal artery occlusion, acute glaucoma, acute eye infections (endophthalmitis, orbital cellulitis, purulent corneal ulcer, acute dacryocystitis, etc.). otorhinolaryngology: severe otorhinolaryngological trauma, foreign body in the airway/esophagus /nasal cavity/external auditory canal, acute laryngitis/laryngeal obstruction, epistaxis/severe postoperative bleeding in otorhinolaryngology, peritonsillar abscess/pharyngeal wall abscess/acute cellulitis in otorhinolaryngology, acute otitis media/otogenic vertigo, etc.	5.00 ± 0.00	0.00
3.1.2 First aid and care of common critical conditions in internal medicine and surgery: respiratory system: asthma, asphyxia, pulmonary embolism, respiratory failure; circulatory system: myocardial infarction, hypertensive emergency, cardiac arrest; endocrine system: hypoglycemia; neurological system: cerebral infarction, cerebral hemorrhage, etc.	4.94 ± 0.24	0.05
3.1.3 Process and management of pre-screening and triage in EENT emergencies	4.89 ± 0.32	0.07
3.1.4 Transfer and handover of EENT emergency patients	4.94 ± 0.24	0.05
3.1.5 Prevention and Control of Hospital Infections in EENT Emergency Rooms	4.78 ± 0.43	0.09
3.2 Specialised Emergency Skills and Procedures	5.00 ± 0.00	0.00
3.2.1 Basic and emergency techniques: sputum aspiration, oxygen administration, intravenous infusion, cardiopulmonary resuscitation (CPR), patient transport, etc.	5.00 ± 0.00	0.00
3.2.2 Specialised operations and emergency care coordination in EENT: EENT specialised procedures: conjunctival sac irrigation, ocular compression bandaging technique, ear compression bandaging technique, tracheostomy dressing change technique; EENT emergency care coordination: nursing practice coordination of anterior and posterior nasal packing, emergency treatment of severe postoperative bleeding, emergency treatment of laryngeal obstruction, coordination of percutaneous tracheostomy, etc.	5.00 ± 0.00	0.00
3.2.3 Use of common emergency equipment/instruments: ECG machine, ECG monitor, defibrillator, simple respirator, CPR machine, infusion pump, micro-infusion pump, electric suction device, ventilator, oxygen cylinder, etc.	5.00 ± 0.00	0.00
3.3 Emergency Humanistic Care and Nursing	4.83 ± 0.38	0.08
3.3.1 Clinical application of humanistic care and empathy in the emergency department	4.39 ± 0.61	0.14
3.3.2 Interpersonal communication methods and techniques in the emergency room	4.72 ± 0.46	0.10
3.3.3 Psychological care methods for emergency patients and their families	4.44 ± 0.62	0.14
3.3.4 Communication methods for terminally ill emergency patients	4.61 ± 0.61	0.13
3.3.5 Self-psychological adaptation for emergency care work	4.50 ± 0.51	0.11
3.4 Education and Research in Emergency Nursing	4.39 ± 0.61	0.14
3.4.1 Common health education methods and techniques in emergencies	4.28 ± 0.57	0.13
3.4.2 Teaching methods and techniques in emergencies	4.33 ± 0.59	0.14
3.4.3 Fundamentals of research in emergency nursing (including literature review, research design, thesis writing methods, etc.)	4.00 ± 0.49	0.12
4. Training Assessment	4.83 ± 0.38	0.08
4.1 Assessment Methods	4.72 ± 0.46	0.10
4.1.1 Assessment methods include theoretical exams, practical exams, and scenario simulation exams.	4.94 ± 0.24	0.05
4.2 Assessment Criteria	4.83 ± 0.38	0.08
4.2.1 Passing scores for theoretical exams, practical exams, and scenario simulation exams (percentile scale, with an average score of not less than 80 points).	4.89 ± 0.32	0.07
4.2.2 Completion of training contents and passing theoretical, practical, and scenario simulation exams.	4.94 ± 0.24	0.05
4.3 Training Effectiveness Evaluation	4.89 ± 0.32	0.07
4.3.1 Pass rate of assessments = number of individuals who passed/total number of training participants.	4.72 ± 0.46	0.10
4.3.2 Trainee satisfaction	4.67 ± 0.49	0.10
4.3.3 Core competency assessment for trainees	4.72 ± 0.46	0.10

## Discussion

### Significance of building a training program for EENT Emergency nurses

The “National Nursing Career Development Plan (2021–2025)” [3] explicitly states that a nurse training system should be established with job demands as its guiding principle and job competency as its core. This system should be aligned with the healthcare needs of the public and the development of nursing disciplines. Special emphasis should be placed on providing job training for nurses in shortage areas, such as emergency and critical care nursing, to enhance their technical expertise in nursing specialties. This study was guided by the core competency standards for emergency nursing and has ultimately developed a training program. This program includes training objectives, specialised training content tailored to EENT, training management, and assessment criteria. This text can serve as a theoretical foundation for the training, managing, and assessing of EENT emergency nurses, promoting the advancement of EENT emergency nursing.

### Reliability of training program for EENT emergency nurses

This study considers the multiple perspectives to develop the training program. In the initial stages of program formulation, the research team conducted a comprehensive review of relevant domestic and international literature, documents, and policies. This study aimed to gain insights into the current state of emergency nursing training for specialised nurses both in China and abroad. The findings will provide a reference for the development of a training program. To ensure that the constructed training program aligns with the current status of EENT emergency nursing training and meets clinical demands, it was refined. During the expert consultation phase, experts with intermediate or higher titles from various provinces and cities, including Shanghai, Shandong, Jiangsu, and Zhejiang, were selected. These experts came from fields such as emergency nursing, nursing management, otolaryngology, and ophthalmology. They provided constructive feedback on the training program, and both rounds of questionnaire surveys achieved a 100% response rate. The selected experts’ high level of engagement demonstrates their strong commitment and expertise in emergency nursing practice and education. The authority coefficient of 0.905 further confirms their extensive experience Additionally, good consensus was achieved among the experts regarding the indicator settings, with all coefficient of variation values being less than 0.20 after two rounds of expert consultation. Therefore, this study’s training program for EENT emergency nurses demonstrates a high degree of scientific rigor and reliability.

### Content analysis of the training program for EENT emergency nurses

The study’s training program comprises four main aspects: training objectives, training management, training contents, and training assessment. These aspects are considered to be relatively comprehensive. The first aid training system for head and neck cancer nurses constructed by Cao et al. [23] only included training objectives and content, lacking two important elements: training management and assessment. The training objectives were guided by core competencies, and a content analysis and deconstruction of the core competencies of emergency nurses were conducted based on the knowledge, abilities, and professional qualities associated with the objectives. (1)Training Objectives: The training objectives consist of three major modules covering knowledge, skills, and professional qualities. There are a total of 16 specific objectives. The emphasis is put on developing core competencies for EENT emergency nurses, including practical abilities, critical thinking skills, and communication skills. In the objective framework for ophthalmic specialty nurse training conducted by Hu et al. [29], the cognitive, skill, and emotional domains were incorporated into the training objectives, Despite the differing titles of the training objectives, the specific training goals were similar. Practical abilities scored higher across various indicators, whereas two indicators related to teaching skills received lower scores. This may reflect the current emphasis on clinical nurse training in China, with room for improvement in teaching skills. Furthermore, ophthalmic nurses themselves are more concerned with their current nursing skills and knowledge, as well as uncertainty in handling patients’ inquiries and practice protocols [19]. (2) Training Management: Training management is a crucial mechanism for organizing and conducting training. It includes criteria for admitting trainees and instructors, training methods, duration, and teaching methods. Clear requirements for the specific implementation and conduct of training are presented to ensure quality management. The ophthalmic specialist nurse training program constructed by Jiang et al. [17] also provided clear explanations of training management content, including the admission of trainees and the duration of training. The criteria for admitting trainees, particularly regarding years of work experience, have sparked debate among experts. After considering the workforce and training status of subspecialty nurses in China, the group set a minimum requirement of at least one year of work experience in EENT for trainees. In addition, the qualifications of the instructor have a significant impact on the effectiveness of the training. However, due to the relatively late start of specialised training for emergency nurses, there is a shortage of instructor resources. Therefore, the group refined the admission criteria for teaching staff based on professional practice, teaching abilities, and professional titles, taking experts’ opinions into account. The teaching staff is required to possess certification as specialised nurses at the municipal level or higher. Additionally, the group discussed and referenced the training duration for other specialised fields in China [30], considering the specialisation and complexity of EENT emergency nursing. A clinical practice duration of 1–3 months has been set to balance in-depth learning and the demanding practical requirements. (3) Training Contents: This study identified four areas of training content based on emergency specialty training. These areas include theoretical knowledge in emergency specialties, skills and procedures, humanistic care and nursing, and research and education. The identified areas provide clear guidance for the future design of training courses. The results indicate that indicators related to theoretical knowledge and specialised skills received high scores (all above 4.70). This highlights the significance of providing clinical nurses with specialised practical skills training. Yang et al. [31] also emphasised the importance of specialised knowledge and skills in the training of ophthalmic specialty nurses. The training content of this study encompassed not only EENT specialised knowledge and skills but also the knowledge required for various acute conditions in the EENT emergency department. This is done to better meet the clinical practical needs. Besides, research capability refers to the ability of nursing personnel to engage in continuous nursing research activities in specialised nursing fields. The research capabilities of clinical nurses not only affect the development of the discipline but also have a direct impact on patient outcomes [32]. This study found that education and research indicators in emergency nursing all scored below 4.40, which may be attributed to the relatively late start of specialised nurse training in China. In addition, subspecialty nurses in China mainly hold college and undergraduate degrees, and their research capabilities are still weak [33]. (4) Assessment and Evaluation: Assessment and evaluation are crucial components of the educational and training process, serving as essential means of measuring the quality of education and training. This study mainly includes assessment indicators such as pass rates in theoretical exams, practical exams, and scenario simulations, as well as trainee core competency assessments and trainee satisfaction, based on experts’ opinions and relevant research [34]. Jiang et al. also included theoretical exams, practical exams, and core competency assessments in the assessment of ophthalmic nurses [17]. However, this study did not include challenging assessment elements such as thesis writing and case reports. The training program constructed through group discussion in this study is primarily intended for EENT emergency specialty nurses, rather than clinical nurse specialists.

### Limitations

Due to limitations in research funding, this study was conducted with interviews only with emergency nurses and nursing educators from one hospital. In the future, the sample size will be expanded to explore the training needs of clinical nurses from a greater number of hospitals in greater depth. The study did not validate the application effect of the training program. In the future, feedback from the trainees will be collected and the training program will be further adjusted and verified to ensure its scientific validity. This process of continuous improvement will be employed to enhance the training program.

## Conclusion

This study has established a basic framework for a training program based on a systematic literature review and theoretical analysis. The training program was refined through conducting interviews with EENT emergency nursing managers, specialised emergency nurses, ophthalmologists, and otolaryngologists, based on the theory of core competencies of emergency nurses. The training program was finalised with input from expert consultations. It encompasses training objectives, training management, training contents, and training assessment. This program can serve as a reference guide for the training of EENT emergency nurses. The next step of this study is to apply the training program among EENT emergency nurses. The program will be further improved according to the feedback from practical implementation.

## Data Availability

All data generated or analysed during this study are included in this published article.

## Abbreviations

EENT: Eye, ear, nose, and throat
